# *POFUT1* as a Promising Novel Biomarker of Colorectal Cancer

**DOI:** 10.3390/cancers10110411

**Published:** 2018-10-30

**Authors:** Julien Chabanais, François Labrousse, Alain Chaunavel, Agnès Germot, Abderrahman Maftah

**Affiliations:** 1 Glycosylation and Cell Differentiation, Limoges University, PEIRENE, EA 7500, F-87060 Limoges cedex, France; julien.chabanais@unilim.fr (J.C.); agnes.germot@unilim.fr (A.G.); 2 Department of Pathology, Limoges University Hospital, 87042 Limoges cedex, France; francois.labrousse@unilim.fr (F.L.); alain.chaunavel@chu-limoges.fr (A.C.)

**Keywords:** POFUT1, colorectal cancer, early detection, biomarker, NOTCH

## Abstract

Background: While protein *O*-fucosyltransferase 1 (*POFUT1*) overexpression has been recently proposed as a potential biomarker for different cancer types, no study was carried out on POFUT1 implication in colorectal cancer (CRC). Methods: Data from 626 tumors and 51 non-tumor adjacent tissues available in FireBrowse had been used in this study. Statistical analyses on *POFUT1* expression and gene copy number, *NOTCH* receptors (main targets of POFUT1 enzymatic activity) expression and association of *POFUT1* and *NOTCH1* expressions with clinical parameters were investigated. Data were completed by POFUT1 histological labeling on six tumor tissues from patients with CRC. Results: We found that *POFUT1* is overexpressed from the stage I (*p* < 0.001) and 76.02% of tumors have a 20q11.21 amplification, associated in 90.13% of cases with a *POFUT1* overexpression, compared to non-tumor adjacent tissues. The *POFUT1* copy number in tumors is mainly between 2 and 3. *POFUT1* is positively correlated with *NOTCH1* (r_s_ = 0.34, *p* < 0.001), *NOTCH3* (r_s_ = 0.087, *p* = 0.0297), and *NOTCH4* (r_s_ = 0.097, *p* = 0.0148) expressions, while negatively correlated with *NOTCH2* expression (r_s_ = −0.098, *p* = 0.0142). *POFUT1* overexpression is markedly associated with rectal location, non-mucinous adenocarcinoma and cancer stages IV and M1. *NOTCH1* overexpression is only associated with rectal location and non-mucinous adenocarcinoma. Conclusion: We conclude that *POFUT1* is overexpressed in CRC from stage I, and its high expression is associated with metastatic process, probably through NOTCH pathway activation. Then, POFUT1 could represent a potential novel biomarker for CRC diagnosis.

## 1. Introduction

Colorectal cancer (CRC) is the third most commonly diagnosed cancer in males and the second in females with 1.65 million new cases and almost 835,000 deaths in 2015 [1]. The majority of CRC (75%) has a sporadic origin but in some cases the origin is related to familial heredity or due to inflammatory bowel diseases [2]. Although the mortality associated with CRC declined over the past decades, identification of new biomarkers for an early diagnosis and the improved treatment of CRC are crucial. Previous studies have demonstrated the association between glycosylation changes and tumorigenesis [3,4]. Glycosylation is the main post-translational modification of proteins. *N*- and/or *O*-glycans play major roles as in protein conformation then modulating their functional activity [5], in ligand-receptor complex formation for cell–cell interactions [6], and in cellular metabolism [7]. Fucose is frequently found as a carbohydrate constituent of *N*-glycans at peripheral positions but also, linked to core *N*-acetylglucosamine. It is involved in selectin-dependent leukocyte adhesion, maternal-fetal interface stability, and formation of Lewis blood group antigen [8,9,10]. In malignant transformation, fucosyltransferases are altered in their expressions and activities. For example, in CRC the α1,6 core-fucosyltransferase encoded by *FUT8* is increased in both enzyme activity and protein expression during malignant transformation [11]. The α1,3/4-fucosyltransferase gene *FUT6* is overexpressed in colorectal tissues where the enzyme functions as a tumor regulator by promoting cell growth, migration, invasion and angiogenesis [12]. *O*-fucosylation is an atypical post-translational modification of proteins catalyzed by two glycosyltransferases, Protein *O*-fucosyltransferase 1 (Pofut1) and Protein *O*-fucosyltransferase 2 (Pofut2). Pofut1 modifies epidermal growth factor-like (EGF-like) domains and Pofut2 modifies thrombospondin repeats (TSR) [13]. Increasing evidences demonstrate the role of Pofut1 in controlling the balance between non-differentiated and differentiated normal cells [14,15]. The human glycoprotein POFUT1 consists of 393 amino acids encoded by a gene located between *PLAGL2* and *KIF3B* on the long arm of chromosome 20, near the centromere [16]. Pofut1 is an ER-resident enzyme [17], which allows fucose addition [18] on S or T included in the C^2^X_4_(S/T)C^3^ consensus motif, where C^2^ and C^3^ are the second and third cysteines of the 6 conserved ones in EGF-like domains. The major known target of Pofut1 is Notch receptor, with four paralogs in human, NOTCH1 to NOTCH4, which contain between 29 and 36 EGF-like domains with 14 to 20 *O*-fucosylation consensus sites [19]. Correct glycosylation of Notch receptors especially their *O*-fucosylation [20] is necessary for subsequent optimal cleavages releasing the NICD (Notch intracellular domain), which translocates into the nucleus to control transcription of target genes [21]. In mice, *Pofut1* knockout is lethal; embryos die at midgestation with severe defects in somitogenesis, cardiogenesis, and neurogenesis, and their phenotype is similar to that of embryos lacking downstream effectors of NOTCH signaling pathway [22]. *POFUT1* knockdown in HEK293T cells induces a 2-fold reduction of the amount of NOTCH1 on the cell surface [23]. Indeed, *O*-fucose addition contributes to EGF-like stabilization, which participates to a novel ER quality control pathway, essential to correct targeting of Notch to the cell membrane and its interaction with ligands [24]. Recent evidences demonstrated NOTCH implication in tumoral pathologies. In T cell acute lymphoblastic leukemia (T-ALL), *NOTCH1* gene is mutated in at least 65% of the cases [25] and an aberrant NOTCH signaling is implicated in this pathologic development process [26]. An ectopic *NOTCH1* expression triggers epithelial-mesenchymal transition in human breast cancer inducing tumor growth and metastasis [27]. In gastric cancer cells, NOTCH pathway activation also induces cell proliferation and metastasis, here through phosphorylated STAT3 and TWIST [28]. In CRC, NOTCH pathway participates to the tumor growth by promoting cell proliferation and inhibiting cell apoptosis [29]. As *O*-fucosylation of NOTCH receptor is necessary for its activation, several cancer studies focused on *POFUT1* expression. A decade ago, the first finding showed a higher expression of *POFUT1* in gliomas compared to normal cells [30]. More recently, *POFUT1* overexpression was also detected in oral squamous cell carcinoma and correlated with an increase of tumor size [31]. In hepatocellular carcinomas, it was associated with a poor prognosis, as it induces an aberrant activation of NOTCH pathway, which promotes cell proliferation, migration and invasion [32]. In gastric cancer, increased *POFUT1* expression is associated with some clinical features such as higher TNM staging and tumoral differentiation states [33]. *POFUT1* gene is localized in the 20q11.21 region, which is frequently amplified in tumor cells as for breast [34] and gastric cancers [35], acute myeloid leukemia [36] and colorectal cancer with poor prognosis [37]. In this last case, a positive correlation is reported between *POFUT1* expression and the copy number of the 20q11-13 amplicon [38]. All these data suggest that POFUT1 could play a significant role in cancer development.

Therefore, we started this study to evaluate *POFUT1* expression in CRC and determine its potential value as a novel diagnostic biomarker for this cancer. Using Firebrowse database, we collected expression data from RNAseq, copy number variation (CNV) of *POFUT1* gene and diverse clinical information. In parallel, based on six different colorectal tumors, we detected POFUT1 and estimated the number of *POFUT1* copies.

## 2. Results

### 2.1. *POFUT1* is Overexpressed in Human Colorectal Cancer Tissues

**Figure 1:**
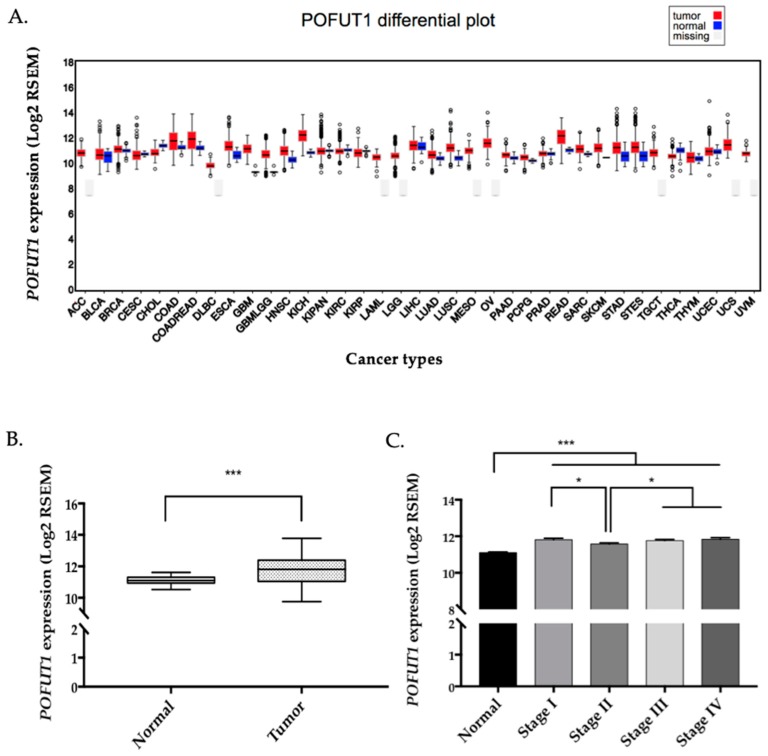
*POFUT1* is overexpressed in most of cancer types especially in colorectal cancer from the first stage. RNAseq data from FireBrowse database show that in 22 cancer types (including COAD and READ), *POFUT1* expression is higher than in the corresponding normal tissues and for 6 cancer types it is the reverse (**A**). Data are missing for nine cancer types. ACC: adrenocortical carcinoma, BLCA: bladder urothelial Carcinoma, BRCA: breast invasive carcinoma, CESC: cervical squamous cell carcinoma and endocervical adenocarcinoma, CHOL: cholangiocarcinoma, COAD: colon adenocarcinoma, COADREAD: colorectal adenocarcinoma, DLBC: lymphoid neoplasm diffuse large B-cell lymphoma, ESCA: esophageal carcinoma, GBM: glioblastoma multiforme, GBMLGG: glioma, HNSC: head and neck squamous cell carcinoma, KICH: kidney chromophobe, KIPAN: pan-kidney cohort, KIRC: kidney renal clear cell carcinoma, KIRP: kidney renal papillary cell carcinoma, LAML: acute myeloid leukemia, LGG: brain lower grade glioma, LIHC: liver hepatocellular carcinoma, LUAD: lung adenocarcinoma, LUSC: lung squamous cell carcinoma, MESO: mesothelioma, OV: ovarian serous cystadenocarcinoma, PAAD: pancreatic adenocarcinoma, PCPG: pheochromocytoma and paraganglioma, PRAD: prostate adenocarcinoma, READ: rectum adenocarcinoma, SARC: sarcoma, SKCM: skin Cutaneous Melanoma, STAD: stomach adenocarcinoma, STES: stomach and esophageal carcinoma, TGCT: testicular germ cell tumors, THCA: thyroid carcinoma, THYM: Thymoma, UCEC: uterine corpus endometrial carcinoma, UCS: uterine carcinosarcoma, UVM: uveal melanoma. COADREAD RNAseq data extracted from FireBrowse database containing 626 CRC and 51 normal adjacent tissues show that *POFUT1* is significantly overexpressed in tumor tissues (**B**) and from the first stage of tumor classification (**C**). For B. and C., bar graph represented mean of log2 RSEM ± SEM. Statistical significance was assessed using a two-tailed Student test; * *p* < 0.05, *** *p* < 0.001.

On a panel of 28 cancer types available in FireBrowse database, *POFUT1* expression is predominantly higher in 22 tumors compared to normal tissues (Figure 1A). COAD (colon adenocarcinoma) and READ (rectum adenocarcinoma) presented the greatest means of log2 RSEM (RNA-Seq by Expectation Maximization) 11.633 and 11.962, respectively for *POFUT1*, only being exceeded in chromophobe kidney carcinoma KICH (12.086). As expected, the COADREAD data, which are a compilation of COAD and READ, showed an increased *POFUT1* expression in tumor compared to healthy tissues.

**Figure 2:**
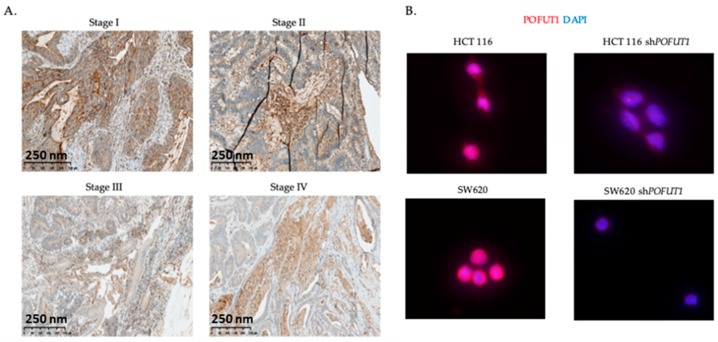

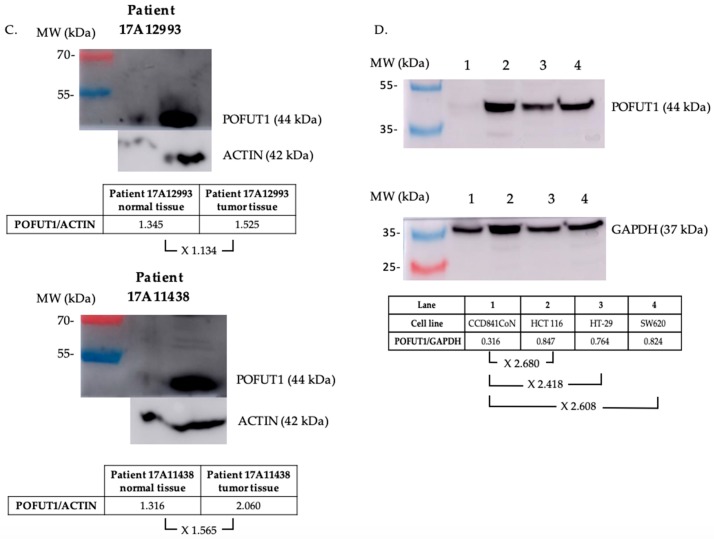
POFUT1 expression in colorectal tissues and cell lines. Immunohistochemistry analysis (**A**) shows that POFUT1 is overexpressed in tumor tissues from first colorectal cancer (CRC) stage. Immunofluorescence labeling of POFUT1 (red), performed on wild-type and *POFUT1* knockdown HCT 116 and SW620 human colorectal cancer cell lines, confirmed the antibody specificity (**B**). Western blot realized on colorectal tissues (**C**) and colorectal CCD841CoN, HCT 116, HT-29 and SW620 cell lines (**D**) validate the POFUT1 overexpression in cancer samples compared to healthy samples.

To investigate in detail *POFUT1* expression in colorectal cancer (CRC), an in silico analysis using the RNAseq data of COADREAD samples extracted from FireBrowse was performed using 626 tumor and 51 adjacent non tumor tissues. *POFUT1* expression is significantly higher in 459 (72.8%) tumor compared to normal tissues (*p* < 0.001) (Figure 1B). The distinction between cancer stages showed a significant (*p* < 0.001) increase in *POFUT1* expression whatever the stage is, therefore at the first signs of the tumor growth (Figure 1C). Stage II presented a lesser amount of *POFUT1* transcripts compared to other stages. POFUT1 immunolabeling performed on tumors representing each CRC pathological stage confirms that POFUT1 is overexpressed in tumor (Figure 2A). To demonstrate anti-POFUT1 antibody (ab74302) specificity, we performed an immunofluorescence detection on two human colorectal cancer cell lines (HCT 116 and SW620) stably transfected or not, with shRNAs targeting *POFUT1*. As shown in Figure 2B, HCT 116 sh*POFUT1* cell line, whose *POFUT1* expression is 30% lesser (quantification by Taqman probe qRT-PCR method), has a lower staining compared to HCT 116. This result is more accentuated with SW620 sh*POFUT1* cell line, which has 60% *POFUT1* expression decrease compared to SW620. As the POFUT1 antibody (ab74302) was ineffective in immunoblotting, we used another antibody raised against Pofut1 and produced in our laboratory [39]. This antibody has been proven in different studies especially in mice [24,40]. Despite a low quality of protein migration due to the Optimal Cutting Temperature (OCT) embedded colorectal tissues, we observed an increase (1.134 and 1.565 fold) of POFUT1 labeling in tumor samples compared to normal tissues (Figure 2C). Such a result was confirmed on human colorectal cancer cell lines HCT 116, HT-29 and SW620 where the expression levels were respectively 2.680, 2.418 and 2.608 fold higher compared to the human embryonic colon cell line CCD841CoN (Figure 2D).

### 2.2. In CRC, 20q11.21 Chromosomic Region is Often Amplified, Which Induced *POFUT1* Copy Number Alteration

**Figure 3:**
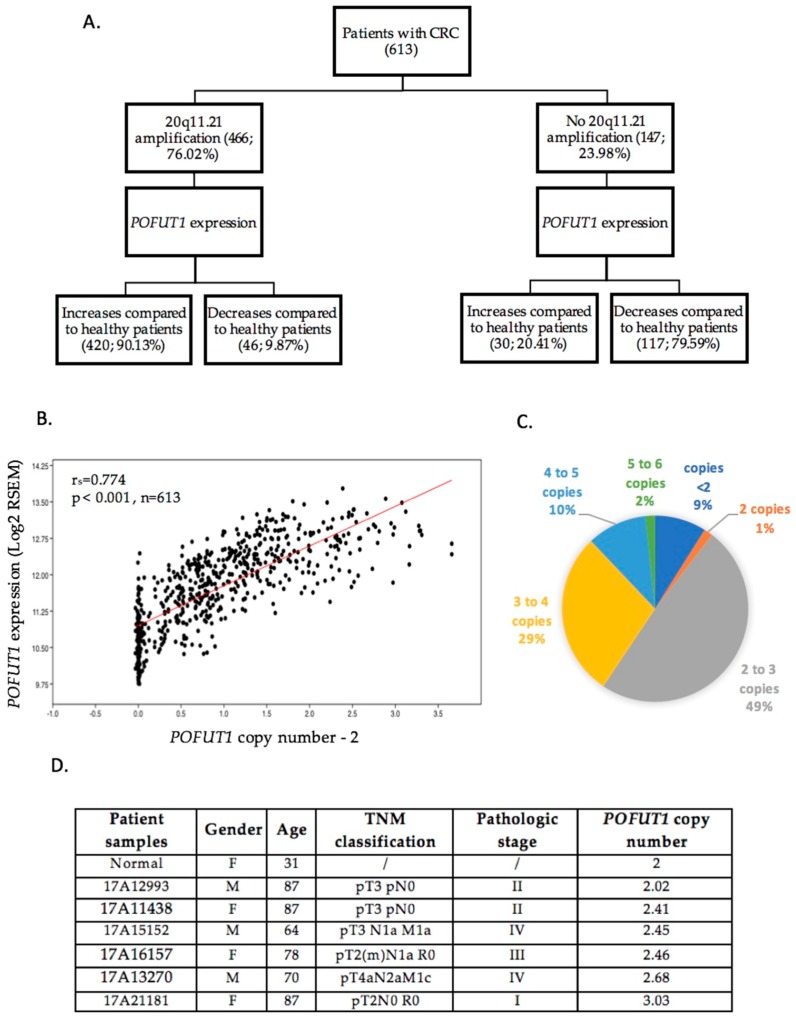
*POFUT1* overexpression is strongly correlated with gene copy number variation. (**A**) Hierarchical analysis of 613 RNAseq data concerning *POFUT1* shows that 76.02% of CRC patients have an amplification of 20q11.21 chromosomic region where *POFUT1* is located. Among them 90.13% have a *POFUT1* overexpression compared to the *POFUT1* mean expression in non-tumor adjacent tissues. (**B**) Spearman Rho correlation analysis in 613 CRC patients shows that *POFUT1* expression is significantly correlated with its copy number. To only view additional copies of *POFUT1* gene, a subtraction of two copies corresponding to a physiological state is applied for each sample. (**C**) CRC patients have in the majority of cases between two and three *POFUT1* gene copies. (**D**) *POFUT1* copy number analysis performed on gDNA extracted from six CRC and one normal tissues shows an increase of *POFUT1* copy number in five CRC cases compared to the normal sample.

To determine if a link exists between *POFUT1* chromosomic region state (20q11.21) and its overexpression, an in silico analysis was performed. The study showed that among 613 patients with CRC, 76.02% had an amplification of the 20q11.21 region, which correlated, in 90.13% of cases, with the increase in *POFUT1* expression compared to healthy patients (Figure 3A). Interestingly, around 80% of CRC patients who had no 20q11.21 amplification presented a lower *POFUT1* expression compared to healthy patients. A significant positive correlation exists between copy number and *POFUT1* expression (r_S_ = 0.774, *p* < 0.001) (Figure 3B), suggesting that *POFUT1* transcript quantity is predominantly due to the gene copy number. Furthermore, copy number analysis of *POFUT1* gene showed that 20q11.21 chromosomic region amplification mostly generates between two and three *POFUT1* copies per genome (49%) and no more than six copies (Figure 3C). Copy number analysis performed on six selected CRC tumors including those immunolabeled by anti-POFUT1 revealed an increase of *POFUT1* copy number in five patients with in majority of cases between 2 and 3 copies, like in bioinformatics analysis (Figure 3D).

### 2.3. Correlation between *POFUT1* and *NOTCH* Receptor Expressions

**Figure 4:**
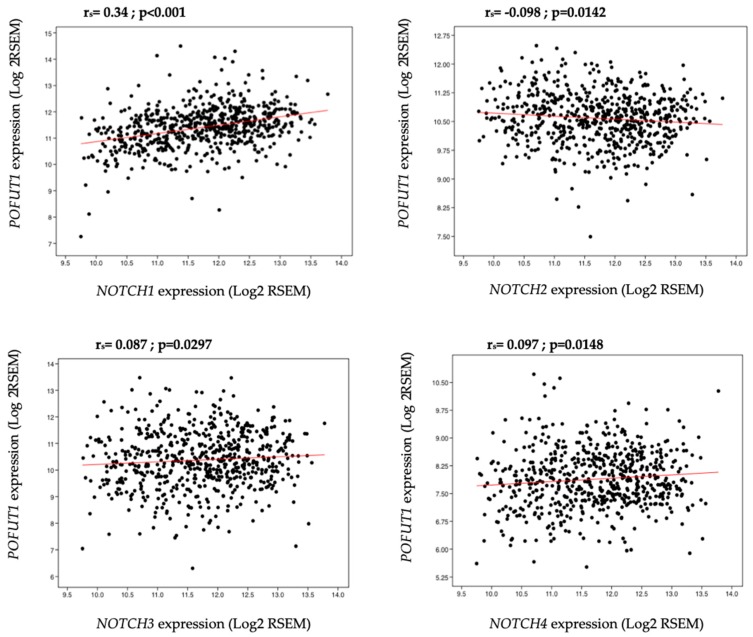
Correlation analysis between *POFUT1* and all *NOTCH* receptor expressions. A total of 626 CRC data for each gene is used for a Spearman Rho correlation. In all of cases, *POFUT1* expression is significantly correlated with *NOTCH* receptor expressions.

Since the cross talk between POFUT1 and NOTCH receptors has been demonstrated in hepatocellular carcinoma and gastric cancer [32,33], a Spearman’s correlation coefficient was used to determine their relationships in CRC. A significant positive correlation was observed between *POFUT1* and *NOTCH1* (r_s_ = 0.34, *p* < 0.001), *NOTCH3* (r_s_ = 0.087, *p* = 0.0297) and *NOTCH4* (r_s_ = 0.097, *p* = 0.0148) receptors (Figure 4). Furthermore, a significant negative correlation was detected between *POFUT1* and *NOTCH2* (r_s_ = −0.098, *p* = 0.0142). Among all correlations, *POFUT1*/*NOTCH1* one was the strongest.

### 2.4. *NOTCH* Signaling Pathway is Deregulated in CRC

**Figure 5:**
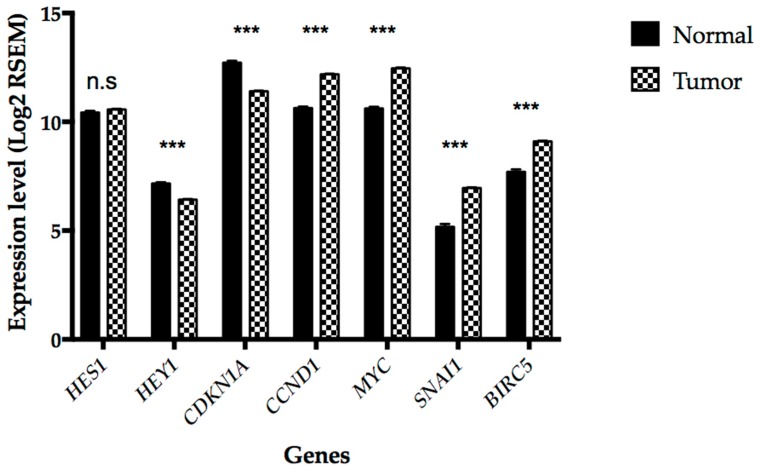
Expression of a set of NOTCH target genes. Data extracted from FireBrowse were obtained from 626 CRC and 51 normal adjacent tissues. The genes related to proliferation (*p21, c-Myc* and *Cyclin D1*), EMT (Epithelial-to-Mesenchymal Transition) process (*Snail 1*) and apoptosis (*Survivin*) are altered in their expressions in the sense of promoting tumor development. *** *p* < 0.001.

Although the expressions of *POFUT1* and *NOTCH1* receptor are significantly and positively correlated, it is necessary to characterize the expression of the NOTCH target genes in order to highlight a potential deregulation of the signaling pathway. We were interested in *HES/HEY* transcription factor gene family especially *HES1* and *HEY1* widely studied in NOTCH pathway analysis, *p21* (*CDKN1A*) and *Cyclin D1* (*CCND1*) that encode cell cycle regulators, *c-Myc* (*MYC*) which is an oncogene, *Snail 1* (*SNAI1*) implicated in EMT and *Survivin* (*BIRC5)* related to apoptosis regulation (Figure 5). All genes, except *HES1*, are significantly modified in their expression levels in tumor compared to normal tissues (*p* < 0.001). *Cyclin D1* and *c-Myc* that induce proliferation, *Snail 1* that promotes EMT and *Survivin* that inhibits apoptosis are overexpressed. *p21*, a negative regulator of cell cycle, and *HEY1* transcription factor mediator of Notch signaling, are downregulated. Taken together these results demonstrate that NOTCH signaling is altered in CRC.

### 2.5. Relationship between *POFUT1*, *NOTCH1* Expressions, and Clinical Features

To further explore the association between *POFUT1* and *NOTCH1* in CRC progression, analysis of their expressions compared to the mean value of healthy patients was studied in the light of various clinical parameters in CRC patients (Table 1 and Table 2). *POFUT1* expression is significantly associated with tumor issue site (*p* = 0.0001), overexpressed in 68.9% of colon and 84.6% of rectum tissues (Table 1). It is linked to pathologic stage (*p* = 0.00019) and markedly overexpressed in 79% of stage I, 63.3% of stage II, 74.9% of stage III and 85.2% of stage IV. *POFUT1* expression is associated with M classification (*p* = 0.01087) and overexpressed in 70.7% of M0 stage and 83.9% of M1 stage. In addition, *POFUT1* is differently expressed according to histological type (*p* = 0.00001) with an overexpression in 75% of colon adenocarcinoma, 30.6% of colon mucinous adenocarcinoma, 88.4% of rectal adenocarcinoma and 46.2% of rectal mucinous adenocarcinoma. However, no correlation was observed between *POFUT1* expression and gender, age, T and N classifications.

*NOTCH1* expression was significantly associated with tumor issue site (*p* = 0.00099) and overexpressed in 70.9% of colon and 84% of rectum tissues (Table 2). It is associated to histological type (*p* = 0.00173) and *NOTCH1* is overexpressed in 73.2% of colon adenocarcinoma, 59.7% of colon mucinous adenocarcinoma, 84.4% of rectal adenocarcinoma and 76.9% of rectal mucinous adenocarcinoma. No correlation was observed between *NOTCH1* expression and gender, age, pathological stage, T, N and M classifications.

In addition, we classified CRC patients into four groups according to their combined expression status of *POFUT1* and *NOTCH1* as follow: high *POFUT1*/high *NOTCH1*, low *POFUT1*/low *NOTCH1*, high *POFUT1*/low *NOTCH1* and low *POFUT1*/high *NOTCH1*. The threshold value which allows to classify individuals in the high and low groups is the mean expression value of *POFUT1* and *NOTCH1* in healthy patients. The associations between these groups and clinical features were analyzed in Table 3. Combined *POFUT1*/*NOTCH1* expressions were significantly associated with tumor issue site (*p* = 0.00004), pathologic stage (*p* = 0.00498) and histological type (*p* < 0.001). It should be noted that in all cases, the majority of CRC are located in high/high group.

## 3. Discussion

Comprehension of mechanisms which initiate tumor development is crucial since an early diagnosis of cancer can trigger treatment and increase the patient chances of recovery. Therefore, research of new potential diagnostic markers of cancer occupies a substantial part in the scientific field. Recently, many studies focused on glycosylation, especially in malignant tumor development [41]. In that context, *O*-fucosylation, linked to expression of Protein *O*-fucosyltransferase 1 (*POFUT1*) and its activity on EGF-like domains, appears promising. POFUT1 adds *O*-fucose on S or T residues within the consensus sequence C^2^X_4_(S/T)C^3^ of EGF-like domains [42] present in some cell surface and secreted proteins [43]. In humans, 87 putative POFUT1 targets had been referenced [44]. Among those, NOTCH receptors are the most described in literature and their *O*-fucosylation was shown to be essential for their interaction with ligands and therefore for NOTCH signaling [24]. POFUT1 and NOTCH cross talk had been described in two cancer types. In breast cancer, an overexpression of *POFUT1* and *NOTCH1* was associated with lymph node metastasis and advanced tumor stage [45]. In hepatocellular carcinoma, *POFUT1* overexpression induced an aberrant activation of NOTCH pathway switching on *HES1*, which in turn promoted migration and cell proliferation [32]. Currently, no study focused on the implication of *POFUT1* in colorectal cancer, although it is a major public health issue. Colorectal cancer is one of the cancers where *POFUT1* is the most overexpressed. Here, bioinformatics combined with immunohistochemistry, western blot and gene copy number analysis had been used as an approach to determine if POFUT1 could be a potential novel CRC biomarker. Among the data of 626 CRC patients available in FireBrowse database, 459 (72.8%) had a *POFUT1* overexpression compared to healthy patients. The overexpression was detected from the first stage of CRC. POFUT1 labeling on CRC biopsies confirmed the overexpression in tumor compared to the adjacent non-tumor tissues. The chromosomic region 20q11.21, where *POFUT1* gene is located, appears to be unstable leading to gene copy number variation, which could explain the expression increase [46]. In the CRC panel, we observed a 20q11.21 amplification in 466 cases over 613 (76.02%), which induces an increase of *POFUT1* gene copy number to 5.7 copies. Our *POFUT1* copy number analysis performed on six CRC tissues follows the same trend as the bioinformatics analysis. As expected, the small sample size does not allow observing the whole range of copy number alterations. A strong positive correlation (r_s_ = 0.774) between *POFUT1* copy number and its expression argues for a direct link as already noticed [38]. It should be noted that the significant decrease of *POFUT1* expression in stage II (Figure 2B) is mainly due to a greater proportion of cases without 20q11.21 chromosomic region amplification (33.9%) compared to other stages (Stage I, 23.1%; Stage III, 18.0%; Stage IV, 13.8%). Significant correlations between *POFUT1* and *NOTCH* receptor expressions were measured with the strongest for *POFUT1*/*NOTCH1* association (r_s_ = 0.34). *POFUT1*, *NOTCH1* and *POFUT1/NOTCH1* high expressions are significantly associated with the tumor issue site, preferentially overexpressed in rectum tissue (84.6%, 84%, 76.1%, respectively). Several other studies highlighted different gene expressions and genetic features associated with carcinogenesis between colon and rectum [47,48]. Interestingly, *POFUT1,* and not *NOTCH1,* expression appears to be significantly associated with M classification. *POFUT1* is predominantly overexpressed in colorectal metastasis (83.9%) and could *O*-fucosylate other protein targets than *NOTCH* receptors, such as *AGRIN* which was shown to enhance tumor progression by activating cell migration and invasion in oral cancer [49]. Overexpression of *POFUT1* and *NOTCH1* is preferentially observed in non-mucinous adenocarcinoma histological type. This observation can be explained by the fact that mucinous adenocarcinoma are characterized by a markedly reduced rate of copy-number aberrations compared to adenocarcinoma [50]. Indeed, in mucinous adenocarcinoma, only 42.6% of cases had an amplified 20q11.21 region. Regarding NOTCH signaling activation, it is known that in tumor tissues a greater activation of NOTCH pathway is involved in cell proliferation and metastasis process [51,52]. In this study on CRC, an increase of NOTCH activation is supported by the higher expression levels of its target genes such as *p21*, *Cyclin D1*, *c-Myc*, *Survivin* and *Snail 1*. However, the expression of *HES1*, a proved Notch signaling downstream target, is not modified in tumor compared to healthy tissues (Figure 5). Nevertheless, studies in relation with *HES1* expression in CRC are controversial [53,54,55] suggesting that it is not a good marker of NOTCH signaling activation in colorectal cancer. We also showed that *HEY1* expression is downregulated although this gene is also known to be activated by Notch signaling [56,57]. As well, conflicting studies showed that this NOTCH target transcriptional factor was overexpressed [58] or non-expressed [59] in colorectal cancer. Surprisingly, significant positive correlations between the expressions of *POFUT1* and *HES1* or *HEY1* are found for healthy tissues and not for tumor ones (Figure S1). It could be explained by the cell heterogeneity of tumors. Therefore, the consequences of *POFUT1* overexpression on NOTCH signaling activation could be opposite depending on the NOTCH target genes. The effect of *POFUT1* overexpression on *O*-fucosylation levels and NOTCH signaling would be cell-type dependent. Overexpression of *POFUT1* most likely does not result in increased *O*-fucosylation of NOTCH receptors. Indeed, in HEK293T cells, most EGF-like repeats containing *O*-fucose consensus sequences are *O*-fucosylated at high stoichiometry degree [60]. If it is the case in colorectal cancer, the overexpression of *POFUT1* may affect the *O*-fucosylation state of other proteins. Nevertheless, it is important to note that in the majority of CRC cases analyzed in the present study, both *POFUT1* and *NOTCH1* are overexpressed (60.5%), suggesting that overexpression of *POFUT1* is necessary to ensure *O*-fucosylation of additional NOTCH receptors in the tumor. Finally, in addition to its *O*-fucosyltransferase activity, it had been demonstrated an independent chaperone function for the POFUT1 orthologue in *Drosophila melanogaster* [61]. However, this additional function is still controversial in mammals [24]. Ajima et al. (2017) [62] showed that in mouse it is not possible to dissociate the possible chaperone contribution from its *O*-fucosyltransferase activity, which could also be the case for human POFUT1. Our study focuses on *POFUT1* expression level related to its copy number determined by 20q11.21 chromosomic region state. Nevertheless we cannot exclude that gene expression level can also be modified by other mechanisms such as mutations within promoter or by miR-34 family regulation [63]. The lack of these informations in the database did not allow us to explore these expression regulatory mechanisms.

## 4. Materials and Methods

### 4.1. The Cancer Genome Atlas Data Analysis

Data for colorectal carcinoma were extracted from FireBrowse database (http://www.firebrowse.org). A total of 626 tumor samples and 51 normal samples were studied. Gene expression levels were merged from COADREAD.uncv2.mRNAseq_RSEM_normalized_log2.txt found in COADREAD.mRNAseq_Preprocess.Level file. Clinical features for each patient were determined from “CLI_years_to_birth”, “CLI_tumor_tissue_site”, “CLI_pathologic_stage”, “CLI_pathology_T_stage”, “CLI_pathology_N_stage”, “CLI_pathology_M_stage”, “CLI_gender” and “CLI_histological_type” extracted from COADREAD-TP.samplefeatures.txt available in COADREAD-TP.Aggregate_AnalysisFeatures.Level metadata. *POFUT1* gene copy number was retrieved from all_data_by_genes.txt provided in COADREAD-TP.CopyNumber_GISTIC2.Level metadata. The presence or absence of 20q11.21 chromosomic region amplification was found in transformed.cor.cli from COADREAD-TP.Correlate_Clinical_vs_CopyNumber-Focal.Level metadata.

### 4.2. Statistical Analysis

Statistical analyses were performed using Past3 3.20 version [64] and GraphPad Prism 7 (GraphPad Software Inc, San Diego, CA, USA). mRNA expression data were referenced as mean ± SEM and a t-Student test was applied to compare values between normal and tumor tissues. Bivariate correlation analysis between *POFUT1* and *NOTCH* receptor mRNA expressions was performed using Spearman’s Rho. Associations between *POFUT1*, *NOTCH1* expressions and clinicopathological parameters were estimated by a Chi-square test. Results were considered statistically significant if the p-value was less than 0.05.

### 4.3. Genomic DNA Extraction and *POFUT1* Copy Number Analysis

Genomic DNA was extracted from normal and tumor tissues with Maxwell^®^ 16 FFPE Plus LEV DNA Purification Kit and Maxwell^®^ 16 IVD device (Promega, Madison, WI, USA) according to the manufacturer’s protocol. Genomic DNA concentration was determined using Quantifluor^®^ ONE dsDNA system (Promega) and measured with Quantus™ Fluorometer (Promega) following manufacturer’s recommendations. Taqman™ copy number assay for *POFUT1* (Hs02487189_cn) and RNAse P reference assays (4403326) were used with Gene Expression Master Mix (Applied Biosystems™, Thermo Fisher Scientific, Waltham, MA, USA), according to product literature. Twenty nanograms of gDNA were run in triplicate on QuantStudio 3 real-time PCR system (Applied Biosystem™). *POFUT1* copy number was estimated using ∆∆Ct method [65].

### 4.4. *POFUT1* Labelling by Immunohistochemistry

Paraffin-embedded blocks of six colorectal adenocarcinomas corresponding to each CRC pathological stage were obtained from the Tumor Bank (CRBiolim) of Limoges University Hospital. Immunohistochemical analysis was performed on five-μm-thick paraffin sections with anti-POFUT1 antibody (1/25, ab74302, Abcam, Cambridge, UK). Slides were automatically processed (Ventana Benchmark ULTRA, Roche, Meylan, France) according to the protocol supplied by the manufacturer. Images were acquired with NanoZoomer RS 2.0 Hamamatsu (Hamamatsu Photonics, Massy, France). All samples were used in accordance with French bioethics laws regarding patient information and consent. Ethics approval (CRB-CESSION-2018-016) was obtained from the “Comité médico-scientifique de la tumorothèque de l’Hôpital Dupuytren”, the bioethics committee of our hospital.

### 4.5. *POFUT1* Labeling by Immunofluorescence

Colorectal cancer cell lines, HCT 116 and SW620 obtained from ATCC and the stably transfected cell lines, HCT 116 sh*POFUT1* and SW620 sh*POFUT1*, created by our team were fixed with 4% paraformaldehyde in PBS for 30 min at room temperature and permeabilized with HEPES Triton buffer (20 mM HEPES, 300 mM sucrose, 50 mM NaCl, 3 mM MgCl2, 0.5% Triton X-100, pH 7.4) for 30 min at 4 °C. After three washes with PBS, non-specific binding sites were saturated for 1 h at room temperature with a blocking solution containing 10% goat serum, 1% BSA, 0.1% Triton X-100 in PBS. After one wash with PBS/0.2% BSA, immunolabeling was performed with anti-POFUT1 (ab74302) antibody diluted at 1:100 in PBS/1% BSA overnight at 4 °C. After washes with PBS and PBS/0.2% BSA/0.1% Tween-20, cells were incubated with the F(ab’)2 fragment of goat anti-rabbit IgG (H + L) secondary antibody Alexa fluo^®^ 546 conjugated (Molecular Probes, Life Technology, Eugene, OR, USA) used at 1:1000 in PBS/1% BSA for 15 min in dark at room temperature. After new washes, nuclei were stained with DAPI (Thermo Fisher Scientific) at 1 µg.mL^−1^ in PBS, 5 min in dark at room temperature. Finally, after three PBS washes, cells were mounted on slides with Fluoromount-G^®^ (SouthernBiotech, Birmingham, AL, USA) and sealed with glass coverslips. We used the MetaMorph^®^ software (Molecular Devices, Sunnyvale, CA, USA) to acquire images with a LEICA microsystem DMI6000B inverted epifluorescence microscope.

### 4.6. Protein Extraction and Western Blot

Total cell protein extracts were prepared by solubilizing tissue or cell pellets (from CCD841CoN, HCT 116, HT-29 and SW620 cell lines obtained from ATCC) in a RIPA lysis buffer (50 mM Tris-HCl, 150 mM NaCl, 1% Triton X-100 (*v/v*), 0.5% sodium deoxycholate (*w/v*), 0.1% sodium dodecylsulfate (*v/v*), pH 8) and a cocktail of protease and phosphatase inhibitors (Roche Applied Science, Mannheim, Germany) for 1 h 30 min at 4 °C. Protein lysates were centrifuged at 12,000× *g* for 20 min at 4 °C, and protein supernatant concentrations were determined using Pierce™ BCA protein assay kit (Thermo Scientific™, Rockford, IL, USA) with bovine serum albumin (BSA) as a standard. Equal amounts of proteins (25 or 50 µg) were resolved by SDS-PAGE using 12% polyacrylamide gels for 1 h at 20 mA. Proteins were transferred onto Amersham™ Protra^®^ premium 0.2 µm nitrocellulose (GE Healthcare, Buckinghamshire, UK) for 1 h 30 min at 0.8 mA/cm^2^. Membranes were blocked with TBS (50 mM Tris, 150 mM NaCl, pH 7.6) supplemented with 0.1% Tween-20 (*v/v*) (TBST) and 5% (*w/v*) non-fat dry milk during 1 h at room temperature. They were incubated with anti-POFUT1 [39], anti-GAPDH (AF5718, R&D Systems, Minneapolis, MN, USA) or anti-Actin (sc-1615, Santa Cruz Biotechnology, Santa Cruz, CA, USA) antibodies, diluted at 1:1000 in TBST, 2.5% (*w/v*) non-fat dry milk overnight at 4 °C. After three washes with TBST, membranes were incubated with secondary antibodies (anti-goat or anti-rabbit HRP-conjugated IgG, Dako, Glostrup, Denmark) diluted at 1:1000 in TBST, 2.5% (*w/v*) non-fat dry milk for 1 h at room temperature. After three washes in TBST, reactive proteins were visualized with ECL™ Prime Western blotting system (GE Healthcare, Uppsala, Sweden). For detection and relative quantification of band intensities, we used Amersham Imager 600 device (GE Healthcare).

## 5. Conclusions

In conclusion, our findings indicated that *POFUT1* is overexpressed in colorectal cancer driven in majority of cases by a 20q11.21 chromosomic region amplification. This aberrant expression may promote carcinogenesis by *NOTCH* pathway activation. Finally, targeting *POFUT1* seems to be a promising strategy for CRC diagnosis.

## Figures and Tables

**Table 1:** Correlation between *POFUT1* expression and clinical parameters in patients with CRC.

Clinical Parameters	N	*POFUT1*		*p* Value
		High (%)	Low (%)	
**Gender**				
Female	290	203 (70.0)	87 (30.0)	0.10206
Male	331	251 (75.8)	80 (24.2)	
**Age (years)**				
≤60	193	146 (75.6)	47 (24.4)	0.38296
>60	426	308 (72.3)	118 (27.7)	
**Tumor Issue Site**				
Colon	454	313 (68.9)	141 (31.1)	**0.00010**
Rectum	163	138 (84.6)	25 (15.4)	
**Pathologic Stage**				
Stage I	105	83 (79.0)	22 (21.0)	**0.00019**
Stage II	229	145 (63.3)	84 (36.7)	
Stage III	179	134 (74.9)	45 (25.1)	
Stage IV	88	75 (85.2)	13 (14.8)	
**Pathology T Stage**				
T1	20	17 (85.0)	3 (15.0)	0.16581
T2	105	84 (80.0)	21 (20.0)	
T3	423	300 (70.9)	123 (29.1)	
T4	70	50 (71.4)	20 (28.6)	
**Pathology N Stage**				
N0	352	245 (69.6)	107 (30.4)	0.05423
N1	150	120 (80.0)	30 (20.0)	
N2	115	85 (73.9)	30 (26.1)	
**Pathology M Stage**				
M0	460	325 (70.7)	135 (29.3)	**0.01087**
M1	87	73 (83.9)	14 (16.1)	
**Histological Type**				
Colon adenocarcinoma	388	291 (75.0)	97 (25.0)	**0.00001**
Colon mucinous adenocarcinoma	62	19 (30.6)	43 (69.4)	
Rectal adenocarcinoma	147	130 (88.4)	17 (11.6)	
Rectal mucinous adenocarcinoma	13	6 (46.2)	7 (53.8)	

Bold values indicate statistical significance.

**Table 2:** Correlation between *NOTCH1* expression and clinical parameters in patients with CRC.

Clinical Parameters	N	*NOTCH1*		*p* Value
		High (%)	Low (%)	
**Gender**				
Female	290	214 (73.8)	76 (26.2)	0.74724
Male	331	248 (74.9)	83 (25.1)	
**Age (years)**				
≤60	193	141 (73.1)	52 (26.9)	0.58601
>60	426	320 (75.1)	106 (24.9)	
**Tumor Issue Site**				
Colon	454	322 (70.9)	132 (29.1)	**0.00099**
Rectum	163	137 (84.0)	26 (16.0)	
**Pathologic Stage**				
Stage I	105	80 (76.2)	25 (23.8)	0.59717
Stage II	229	163 (71.2)	66 (28.8)	
Stage III	179	135 (75.4)	44 (24.6)	
Stage IV	88	68 (77.3)	20 (22.7)	
**Pathology T Stage**				
T1	20	11 (55.0)	9 (45.0)	0.16062
T2	105	79 (75.2)	26 (24.8)	
T3	423	313 (74.0)	110 (26.0)	
T4	70	56 (80.0)	14 (20.0)	
**Pathology N Stage**				
N0	352	257 (73.0)	95 (27.0)	0.66047
N1	150	114 (76.0)	36 (24.0)	
N2	115	88 (76.5)	27 (23.5)	
**Pathology M Stage**				
M0	460	335 (72.8)	125 (27.2)	0.41731
M1	87	67 (77.0)	20 (23.0)	
**Histological Type**				
Colon adenocarcinoma	388	284 (73.2)	104 (26.8)	**0.00173**
Colon mucinous adenocarcinoma	62	37 (59.7)	25 (40.3)	
Rectal adenocarcinoma	147	124 (84.4)	23 (15.6)	
Rectal mucinous adenocarcinoma	13	10 (76.9)	3 (23.1)	

Bold values indicate statistical significance.

**Table 3:** Correlation between *POFUT1/NOTCH1* expressions and clinical parameters in patients with CRC.

Clinical Parameters	N	*POFUT1/NOTCH1*				*p* Value
		High/High (%)	Low/Low (%)	High/Low (%)	Low/High (%)	
**Gender**						
Female	290	169 (58.3)	42 (14.5)	34 (11.7)	45 (15.5)	0.4020
Male	331	205 (61.9)	37 (11.2)	46 (13.9)	43 (13.0)	
**Age (years)**						
≤60	193	116 (60.1)	22 (11.4)	30 (15.5)	25 (13.0)	0.56050
>60	426	258 (60.6)	56 (13.1)	50 (11.7)	62 (14.6)	
**Tumor Issue Site**						
Colon	454	248 (54.6)	67 (14.8)	65 (14.3)	74 (16.3)	**0.00004**
Rectum	163	124 (76.1)	12 (7.4)	14 (8.6)	13 (8.0)	
**Pathologic Stage**						
Stage I	105	68 (64.8)	10 (9.5)	15 (14.3)	12 (11.4)	**0.00498**
Stage II	229	123 (53.7)	44 (19.2)	22 (9.6)	40 (17.5)	
Stage III	179	107 (59.8)	17 (9.5)	27 (15.1)	28 (15.6)	
Stage IV	88	62 (70.5)	7 (8)	13 (14.8)	6 (6.8)	
**Pathology T Stage**						
T1	20	10 (50.0)	2 (10.0)	7 (35.0)	1 (5.0)	0.09107
T2	105	68 (64.8)	10 (9.5)	16 (15.2)	11 (10.5)	
T3	423	250 (59.1)	60 (14.2)	50 (11.8)	63 (14.9)	
T4	70	43 (61.4)	7 (10.0)	7 (10.0)	13 (18.6)	
**Pathology N Stage**						
N0	352	204 (58.0)	54 (15.3)	41 (11.6)	53 (15.1)	0.42789
N1	150	97 (64.7)	13 (8.7)	23 (15.3)	17 (11.3)	
N2	115	70 (60.9)	12 (10.4)	15 (13.0)	18 (15.7)	
**Pathology M Stage**						
M0	460	266 (57.8)	66 (14.3)	59 (12.8)	69 (15.0)	0.07716
M1	87	61 (70.1)	8 (9.2)	12 (13.8)	6 (6.9)	
**Histological Type**						
Colon adenocarcinoma	388	235 (60.6)	48 (12.4)	56 (14.4)	49 (12.6)	**1.5335 × 10^−15^**
Colon mucinous adenocarcinoma	62	12 (19.4)	18 (29.0)	7 (11.3)	25 (40.3)	
Rectal adenocarcinoma	147	116 (78.9)	9 (6.1)	14 (9.5)	8 (5.4)	
Rectal mucinous adenocarcinoma	13	6 (46.2)	3 (23.1)	0 (0)	4 (30.8)	

Bold values indicate statistical significance.

## Supplementary Materials

The following are available online at http://www.mdpi.com/2072-6694/10/11/411/s1, Figure S1: Correlation analysis between *POFUT1* and *HES1*, *HEY1* transcription factor expressions.
