# Supplementary material for: POFUT1 as a Promising Novel Biomarker of Colorectal Cancer
Source: Cancers (Basel). 2018 Oct 30;10(11):411. doi: 10.3390/cancers10110411 (PMC6266312; doi:10.3390/cancers10110411)
Supplement: Supplementary file 1 [file cancers-10-00411-s001.zip › cancers-367394-SI.pdf]

Cancers 2018, 10, 411; doi:10.3390/cancers10110411 www.mdpi.com/journal/cancers

cancers

Supplementary Materials: *POFUT1* as a Promising Novel Biomarker of Colorectal Cancer

Julien Chabanais, François Labrousse, Alain Chaunavel, Agnès Germot, Abderrahman Maftah

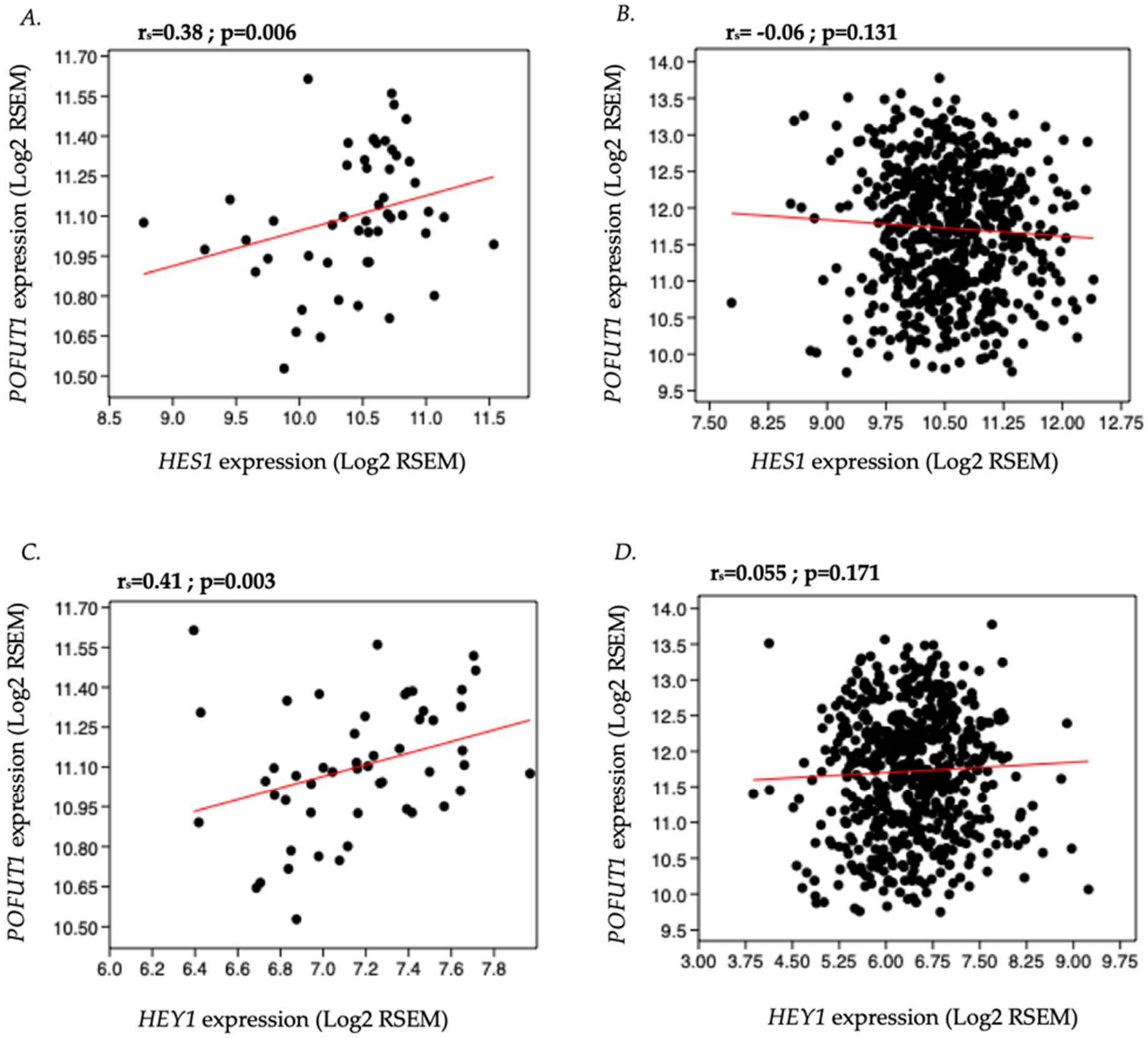

Figure S1. Correlation analysis between POFUT1 and HES1, HEY1 transcription factor expressions**.** A total of 626 CRC data for each gene is used for a Spearman Rho correlation. POFUT1 expression is significantly correlated with HES1 and HEY1 expressions only in healthy tissues, (A) and (C). In tumor tissues no correlation is detected, (B) and (D).
